# Supplementary material for: Complex‐centric proteome profiling by SEC‐SWATH‐MS
Source: Mol Syst Biol. 2019 Jan 14;15(1):e8438. doi: 10.15252/msb.20188438 (PMC6346213; doi:10.15252/msb.20188438)

TNF−alpha/NF−kappa B signaling complex (CHUK, BTRC, NFKB2, PPP6C, REL, CUL1, IKBKE, SAPS2, SAPS1, ANKRD28, REL
Annotated subunits: 12 Subunits with signal: 10
Max. coeluting subunits: 2 Max. completeness: 0.17

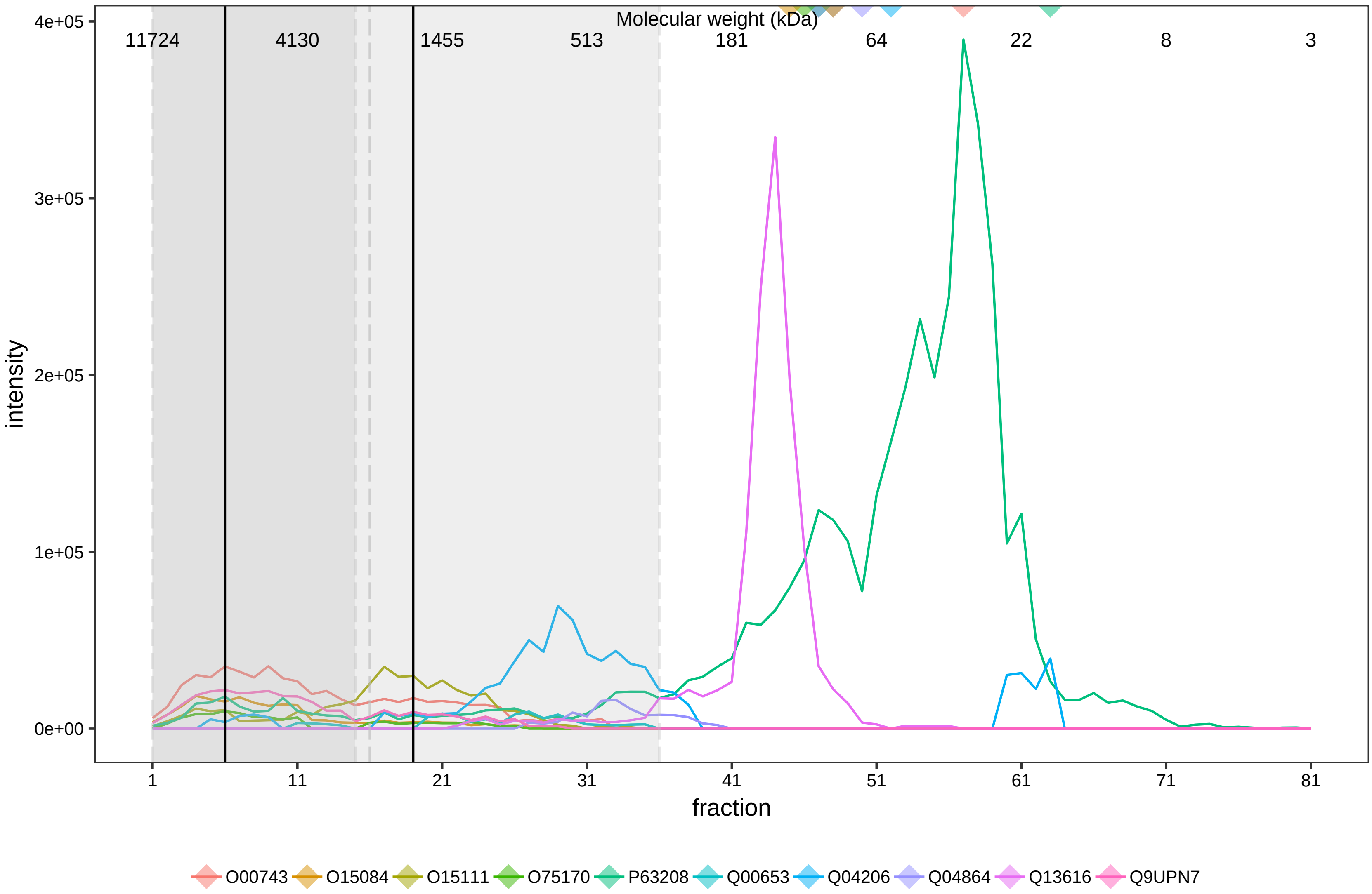

Supplement: Supplementary file 6 — Dataset EV5 [file MSB-15-e8438-s006.zip › feature_plots_corum/5196.pdf]
